# *Neospora caninum* infection and reproductive disorders in Creole goats from southern Peru: A Bayesian mediation analysis across agroecological zones

**DOI:** 10.14202/vetworld.2026.1838-1849

**Published:** 2026-05-10

**Authors:** Jhony Soca, Marisela Trillo-Salvador, Justo Valdivia-Zevallos, Juan Canchino-Gutierrez, Emmanuel Alexander Sessarego, Edwin Mendoza-Alacute, Victor Temoche-Socola, Juancarlos Cruz-Luis, Danny Julio Cruz

**Affiliations:** 1 Estación Experimental Agraria Chincha, Instituto Nacional de Innovación Agraria, Ica 11770, Peru; 2 Escuela de Medicina Veterinaria y Zootecnia, Universidad Privada San Juan Bautista, Ica 11004, Peru; 3 Dirección de Servicios Estratégicos Agrarios, Instituto Nacional de Innovación Agraria, Lima 15024, Peru; 4 Estación Experimental Agraria Canaán, Instituto Nacional de Innovación Agraria, Ayacucho 05007, Peru; 5 Departamento de Producción Animal, Facultad de Agronomía, Universidad de Buenos Aires, Buenos Aires 1417, Argentina

**Keywords:** agroecological zones, Bayesian mediation, Creole goats, *Neospora caninum*, prevalence, reproductive disorders, risk factors, southern Peru

## Abstract

**Background and Aim:**

Neosporosis, caused by *Neospora caninum*, is an important infectious cause of reproductive failure in ruminants, yet its epidemiological role in goats from hyper-arid coastal ecosystems remains poorly understood. In southern Peru, Creole goat production is predominantly extensive, with limited sanitary control and frequent dog–livestock interactions, which may facilitate parasite transmission. This study aimed to estimate the prevalence of *N. caninum* and evaluate its association with reproductive problems in Creole goats, considering age and agroecological zone within a Bayesian mediation framework.

**Materials and Methods:**

A cross-sectional analytical study was conducted between March and June 2025 in the Ica region of southern Peru. A total of 182 female goats from 28 herds with a history of reproductive disorders were included. Serum samples were analyzed using a competitive enzyme-linked immunosorbent assay to determine *N. caninum* serostatus. Reproductive problems, defined as abortion or the birth of weak offspring, were considered the outcome variable. Associations among age, geographic zone, infection status, and reproductive problems were assessed using a Bayesian mediation model to estimate direct, indirect, and total effects.

**Results:**

The prevalence of *N. caninum* was higher in Zone 1 (25.78%; 95% confidence interval [CI]: 18.57–33.58) than in Zone 2 (11.11%; 95% CI: 4.08–20.38). Seropositive goats exhibited a significantly higher likelihood of reproductive problems (odds ratio = 6.49; 95% highest posterior density: 1.76–16.11). Reproductive disorders were more frequent in Zone 2 despite its lower seroprevalence, indicating the influence of non-infectious factors. Age showed a significant positive association with reproductive problems, reflecting cumulative physiological and environmental stress. Mediation analysis revealed that *N. caninum* acted as a direct risk factor rather than a significant mediator of age- or zone-related effects.

**Conclusion:**

*N. caninum* infection is strongly associated with reproductive problems in Creole goats from southern Peru and primarily functions as an individual-level risk factor. Geographic and age-related effects appear to operate largely through direct pathways independent of infection status. These findings highlight the multifactorial nature of reproductive disorders and underscore the importance of integrating infection control with nutritional and management strategies in extensive goat production systems.

## INTRODUCTION

Goat husbandry is a key livelihood strategy in arid and semi-arid regions, where other livestock species face severe adaptive constraints [1, 2]. In these environments, goats are favored for their efficient use of scarce forage, tolerance to heat stress, and adaptability to extensive management systems, which are particularly relevant in arid coastal ecosystems [2]. On the southern coast of Peru, especially in the Ica region, goat production is predominantly extensive and represents an essential socioeconomic activity for small-scale producers [3]. Creole goats constitute an important source of milk and meat, accounting for up to 4.17% of the national herd in 2021 [4]. Nevertheless, reproductive inefficiency remains one of the main constraints affecting productivity in these systems, leading to substantial economic losses [5].

Several infectious agents, including *Brucella melitensis*, *Toxoplasma gondii*, and *Neospora caninum*, have been implicated in the etiology of reproductive disorders in goats [6]. *Toxoplasma gondii* and *B. melitensis* are currently under passive surveillance by the Servicio Nacional de Sanidad Agraria in the Ica region, with no positive cases reported in recent years. In contrast, *N. caninum*, an obligate intracellular protozoan recognized as a major cause of abortion in cattle, has also been reported in goats, where it can induce embryonic resorption, late-term abortion, premature birth, and weak offspring [5, 7, 8]. The heteroxenous life cycle of *N. caninum* involves canids, particularly domestic dogs, which shed oocysts into the environment. These oocysts are ingested by intermediate hosts such as goats, in which infection remains viable for many years through the formation of bradyzoite-containing tissue cysts [9]. In addition, the parasite can be transmitted vertically from infected dams to their offspring through transplacental passage, allowing infection to persist across several consecutive generations within the herd [10].

Although the global distribution of *N. caninum* is well documented, available data in goats remain limited and highly variable, with reported prevalence ranging from 1% to >12% depending on the region and farming conditions [11]. In South America, relatively high prevalence rates have been reported [11]. In Peru, evidence is scarce: one study in Piura reported a seroprevalence of 3.32% [12], whereas another conducted in the tropical dry forest of Utcubamba found rates of 6.21% and 7.21% [13]. Importantly, these studies were limited to northern regions characterized by tropical and dry forest agroecosystems. To date, no investigations have assessed the prevalence of *N. caninum* in Creole goats from southern Peru, nor its potential role in reproductive failures in hyper-arid desert-coastal agroecosystems, which differ markedly in climate, forage availability, and production systems. In this context, the present study represents the first investigation of *N. caninum* infection in goats from a hyper-arid coastal ecosystem, revealing epidemiological patterns that differ substantially from those reported in tropical dry forest or high-altitude environments.

This knowledge gap is particularly relevant because goat production systems in Ica are characterized by extensive management, limited sanitary control, prolonged animal retention, and frequent dog–livestock interaction, all of which may favor parasite transmission [14]. Given the established role of *N. caninum* in reproductive failure, a mediation framework is biologically plausible for exploring whether infection status partially mediates the effects of age and geographical area on reproductive problems. Therefore, this study aimed to evaluate the association between age and geographical area and reproductive problems in Creole goats from the southern coast of Peru, considering *N. caninum* infection as a potential mediating factor.

## MATERIALS AND METHODS

### Ethical approval

All procedures were approved by the Ethics Committee of the Universidad Privada San Juan Bautista under certificate No. 307-2025-CIEI-UPSJB (January 31, 2025), in compliance with institutional regulations and established standards for animal welfare and scientific research. Producer participation was voluntary, and informed consent was obtained verbally and duly documented, ensuring the confidentiality of the information provided at all times.

### Study period and location

**Figure 1:**
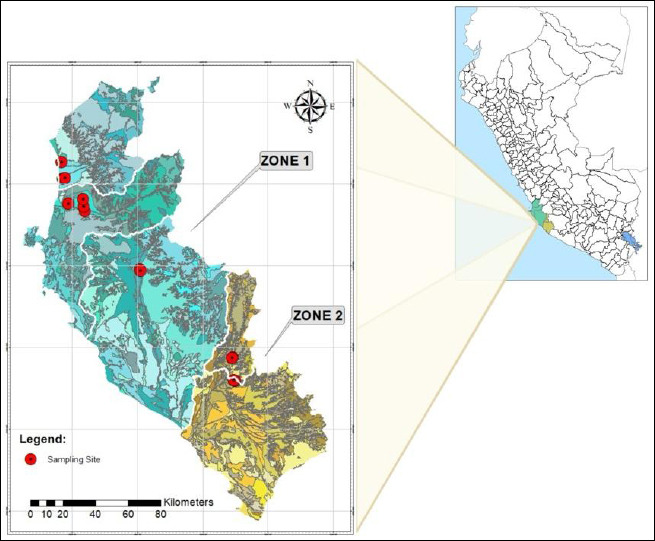
Geographic location of the study area and distribution of goat herds on the southern coast of Peru (Ica region). Zone 1 includes Chincha, Pisco, and Ica provinces, while Zone 2 comprises Palpa and Nazca. The map is projected in UTM Zone 18S (WGS 84) and covers an extent from 373,647 to 480,020 m E and 8,379,754 to 8,513,292 m N [15].

A cross-sectional analytical study was conducted between March and June 2025 in the Ica region (time of parity), on the southern coast of Peru (Figure 1).

**Figure 2:**
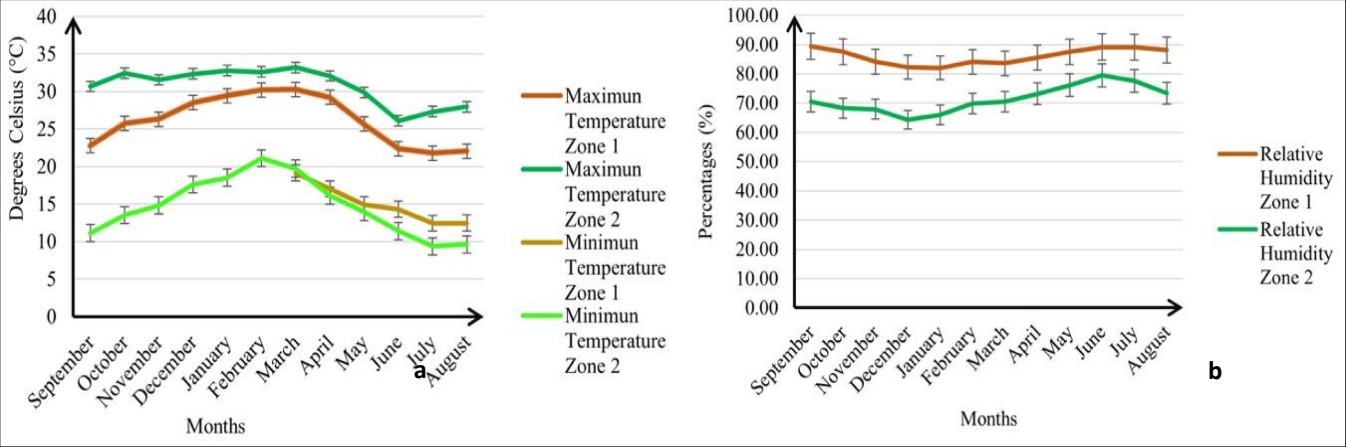
(a) Maximum and minimum temperatures in the study areas. (b) Relative humidity in the study areas. The daily temperature (°C) and relative humidity (%) values were averaged across the three stations in Zone 1 and the two stations in Zone 2 to generate the summary statistics and graphical representations shown in Figure 2.

Two operational zones were evaluated: Zone 1: This zone included herds from the provinces of Chincha, Pisco, and Ica; characterized by a hot arid climate with low rainfall, high solar radiation, temperatures ranging from 12°C to 30°C, and high relative humidity from 82% to 89% [16], as shown in Figures 1 and 2. The area corresponds to coastal valleys with predominantly sandy to sandy-loam soils of alluvial origin, associated with irrigation. Feeding mainly relied on grazing alfalfa and crop residues, such as maize stover, lima bean pods, squash, chickpeas, and sweet potato vines. Zone 2: Included herds from the provinces of Palpa and Nazca and is characterized by a warm arid desert climate, with extremely low rainfall, high solar radiation, and temperatures ranging from 9°C to 33°C, as well as moderate relative humidity from 64% to 79% [16], as shown in Figures 1 and 2. Desert plains with sandy or stony soils and limited natural vegetation dominate the area. Goat feeding relied primarily on browsing native shrubs and trees adapted to arid conditions, such as huarango (*Prosopis limensis*), palo verde (*Parkinsonia praecox*), overo (*Cordia lutea*), and faique (*Vachellia macracantha*, commonly known as espino), primarily along riparian zones with limited access to agricultural residues.

Climate data from 2024 and 2025 from the same stations were used to contextualize the study period within the region’s typical seasonal patterns [16].

### Production systems and farming

In both zones, goat production was predominantly conducted under extensive systems, with herd sizes ranging from approximately 100 to 150 animals. Reproduction is seasonal, with mating occurring from November to January and kidding between April and June, according to the producers’ production calendars [14]. Production is mainly oriented toward milk and its derivatives, as well as the sale of suckling kids (≤25 days old) for meat. The productive lifespan of females generally extended to the fourth and fifth kidding in exceptional cases.

During the humid period, most producers practice seasonal transhumance to lomas (fog-fed hills), where animals graze on native vegetation. This combination of extensive management, seasonal transhumance, reliance on native forage, and limited health interventions defines an epidemiological context that differs markedly from the more sedentary, semi-intensive, or confined goat production systems commonly described in other South American studies. Health management included annual deworming, typically during the postpartum period or when clinical signs are evident, using anthelmintics such as triclabendazole, albendazole, or fenbendazole. In addition, most production units kept between four and six dogs per herd, with no regular deworming programs; this information is recorded only for descriptive purposes.

### Selection of the sampling animals

All sampled animals were from goat-only production systems. Twenty-eight goat herds located in the two study area zones were selected based on a history of reproductive problems, such as repeated estrus cycles, abortions at any stage of gestation, and stillbirths or births of weak live offspring, according to records from the Servicio Nacional de Sanidad Agraria.

For the study, only females from these herds aged between 1 and 6 years who had previously experienced specific reproductive events, defined as abortion or the birth of weak live kids during the kidding period, occurring within the sampling months, were included. Selection was carried out by intentional sampling within each herd, validated against producers’ records, and included only females that had exhibited these events. The final sample comprised 182 female goats. In Zone 1, 128 animals were included, with a mean age of 3.17 ± 1.17 years and a mean body weight of 31.8 ± 2.2 kg; parity ranged from zero to six. In Zone 2, 54 animals were included, with a mean age of 1.92 ± 0.78 years and a mean body weight of 31.2 ± 2.0 kg; parity ranged from zero to four. Values are expressed as mean ± standard deviation (SD). Animal distribution between zones was determined by herd availability and producer accessibility. Table 1 summarizes the general characteristics of the goats sampled by zone, as well as the occurrence of reproductive problems (abortions and weak offspring) and animals without a history of reproductive disorders.

**Table 1:** General characteristics of the Creole goats sampled according to zone and province in the Ica region, Peru’s southern coast.

Zone	Province	Herds	Minimum	Maximum	Mean/Herds	Abortion	Weak offspring	None	Total
Zone 1	Chincha	8	4	5	5.0	16	9	15	40
	Pisco	10	5	11	6.8	48	5	15	68
	Ica	3	6	8	6.67	8	0	12	20
Zone 2	Palpa	4	6	10	7.75	21	3	7	31
	Nazca	3	4	8	5.75	11	0	12	23
Total		28							182

### Blood sample collection

Blood was collected by jugular venipuncture in the morning (07:00–09:00 h) using 20-gauge needles and 6 mL Vacutainer tubes without anticoagulant (Becton Dickinson and Company, New Jersey, USA). Samples were initially stored at room temperature (18°C–25°C), then refrigerated (4°C–8°C), and subsequently transported to the Laboratorio de Sanidad Animal, Facultad de Medicina Veterinaria y Zootecnia, Universidad Privada San Juan Bautista for serum separation. Serum was obtained using a Centurion centrifuge (model C206A) and stored at −20°C until analysis.

### Serological analysis

Antibodies against *N. caninum* were detected using a competitive enzyme-linked immunosorbent assay with the commercial kit ID Screen® *N. caninum* Competition (IDvet, Montpellier, France), following the manufacturer’s instructions. Subsequent mentions are written as *N. caninum*. The absorbance was measured with a Bio-Tek® 800™ TS microplate reader (Bio-Tek Instruments, Vermont, USA), and the data were processed with Gen5™ software (Bio-Tek Instruments). The positive, negative, and cutoff controls provided with the kit were included in each run [17].

Samples were categorized according to the manufacturer’s sample-to-negative control ratio (S/N%) criteria as positive (S/N% ≤ 50%), doubtful (50%–60%), or negative (S/N% > 60%). Doubtful samples were retested in duplicate and reclassified as positive or negative for subsequent analyses.

Independent validation studies of the ID Screen® *N. caninum* Competition enzyme-linked immunosorbent assay report high diagnostic agreement with confirmatory immunoblot, with relative sensitivities of approximately 92.9% and specificities of approximately 97.4% in serum samples, supporting its reliability for serological screening in small ruminants [17].

To ensure analytical reliability, the serum samples were stored at −20°C and transported under cold-chain conditions using insulated containers with refrigerants and temperature monitoring. The transfer of the 182 samples to the Laboratorio de Microbiología y Parasitología, Universidad Nacional Mayor de San Marcos, was completed within 5 h, with continuous temperature control throughout transport.

### Statistical analysis

The prevalence was estimated using the method described by Rafiq [18].


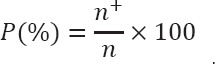


where *P(%)* represents the prevalence expressed as a percentage, n^+^ is the number of animals that tested positive for the evaluated event (*N. caninum* infection or presence of reproductive problems), and *n* is the total number of animals assessed in each study group. Prevalence estimates are presented with their 95% confidence intervals (95% CI), calculated using the normal approximation method for proportions to reflect the uncertainty of the estimates.

**Figure 3:**
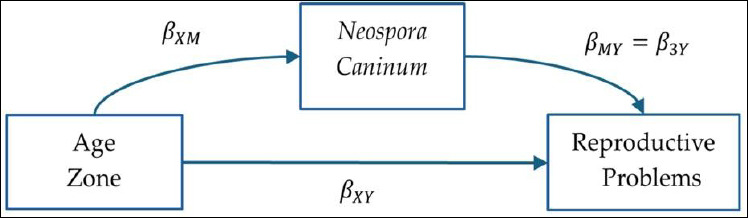
Proposed causal structure for evaluating the effect of covariates X (age or zone) on reproductive problems Y, with *N. caninum* serostatus as the mediating variable M. Arrows represent coefficients on the logit scale of the Bayesian model: a denotes the effect of X on M, *b* the effect of M on Y adjusted for X, and *c*′ the direct effect of X on Y adjusted for M.

A simple logistic regression would estimate overall associations but would not allow separation of direct effects from those operating through *N. caninum*, preventing simultaneous evaluation of the associations of zone and age with the presence of reproductive problems. Therefore, a Bayesian model with a partial mediation structure was applied, with *N. caninum* serostatus as a potential mediating variable, as illustrated in Figure 3.

The model comprised two logistic equations: one for the mediating variable (*N. caninum* serostatus) and the other for the clinical outcome (reproductive problems):

Mediator model (N. caninum serostatus):


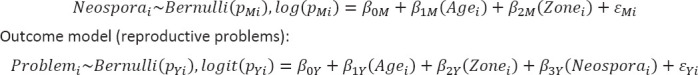


where P(M_i_=1)represents the probability that the i^th^ individual is seropositive for *N. caninum*, and P(Y_i_=1) represents the probability that the same individual presents reproductive problems. α_M_ and α_Y_ are the intercepts of the mediator and outcome equations, respectively. β_1M_ and β_2M_ describe the effects of age and zone on *N. caninum* serostatus, whereas β_1Y_, β_2Y_ and β_3Y_ describe the effects of age, zone, and *N. caninum* serostatus on reproductive problems. All coefficients are expressed on the logit scale.

The Bayesian framework specifies explicit prior distributions. Intercepts in both equations were assigned a Student-t distribution with 3 degrees of freedom, a mean of 0, and a scale of 2.5, and were considered weakly informative on the logit scale. Regression coefficients associated with the covariates (age, zone, and serostatus) were estimated using flat, non-informative priors, allowing inference to be driven primarily by the observed data.

Both equations were estimated simultaneously in the arms package, version 2.23.0 [19], using Hamiltonian Monte Carlo sampling with the No-U-Turn Sampler. Four chains were run for 200,000 iterations each, and the first 8,000 were discarded as warm-up. This yielded 768,000 posterior samples per parameter, assuming independence of the residuals between the two equations. A relatively large number of iterations was used to ensure stable estimation of posterior distributions, particularly for indirect effects derived as products of regression coefficients in the mediation structure.

Convergence was assessed using multiple criteria. The potential scale reduction factor (Rhat statistic) was equal to 1.00 for all parameters, and visual inspection of trace plots indicated adequate mixing and stable exploration of the posterior space. Effective sample sizes were evaluated for both the bulk and tail of the posterior distributions, with all parameters exhibiting large values, indicating efficient sampling and low autocorrelation.

Model adequacy was further evaluated using posterior predictive checks. For both equations of the joint mediation model, the observed data patterns were consistently within the posterior predictive distributions, with no evidence of systematic model misfit.

Inferences were based on posterior means and 95% highest posterior density intervals. Finally, direct effects, indirect effects, and total effects were computed using posterior samples of the parameters obtained from the models, as follows:

Indirect effect = a × b

Direct effect = c ′

Total effect = c ′ + (a × b)

All statistical analyses were conducted using R version 4.5.1 [20].

## RESULTS

### Prevalence of *N. Caninum* infection

**Figure 4:**
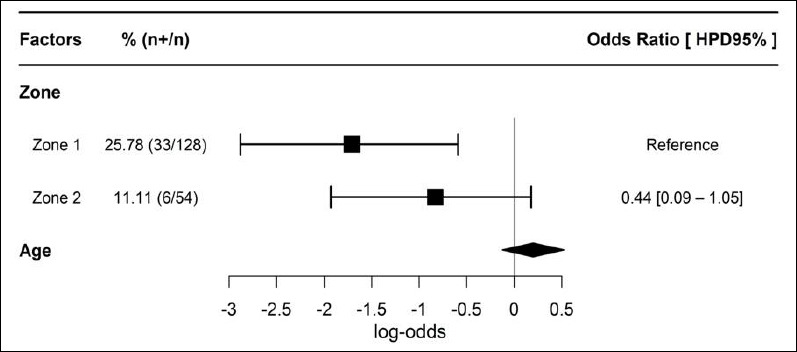
Log-odds and odds ratios of *N. caninum* serostatus by zone and age in Creole goats, obtained from the Bayesian mediator submodel. Squares represent posterior means and 95% highest posterior density (HPD) intervals for zone effects, whereas diamonds represent the posterior mean and 95% HPD interval for the continuous covariate age on the log-odds scale. The vertical line at 0 represents the null effect (odds ratio = 1). Odds ratios (OR = exp[log-odds]) and their corresponding 95% HPD intervals are presented for interpretation. The effect of age is expressed per one unit of increase, where n+ denotes the number of seropositive animals, and n is the total number of animals evaluated per category.

The proportion of animals testing positive for *N. caninum* was higher in Zone 1 than in Zone 2 (25.78%; 95% CI: 18.57–33.58) (11.11%; 95% CI: 4.08–20.38). Adjusted for age, the Bayesian model estimates yielded probabilities of 24.1% (95% highest posterior density (HPD): 16.53–32.10) for Zone 1 and 12.4% (95% HPD: 4.08–23.00) for Zone 2, as shown in Figure 4.

### Frequency of reproductive problems (%)

**Figure 5:**
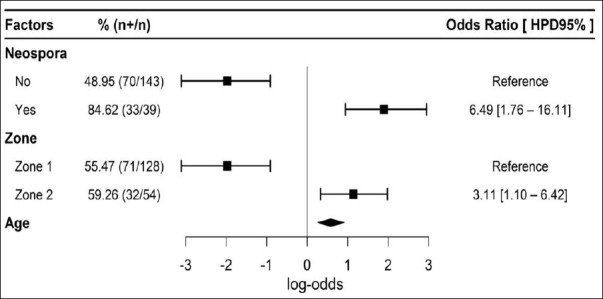
Log-odds and odds ratios of reproductive problems according to *N. caninum* serostatus, zone, and age in Creole goats, obtained from the mediation analysis’s Bayesian outcome submodel. Squares represent posterior means and 95% highest posterior density (HPD) intervals for categorical predictors (*N. caninum* serostatus and zone), whereas diamonds represent the posterior mean and 95% HPD interval for the continuous covariate age on the log-odds scale. The vertical line at 0 represents the null effect (odds ratio = 1). Odds ratios (OR = exp[log-odds]) and their corresponding 95% HPD intervals are presented for interpretation. The effect of age is expressed per one unit of increase, where n+ denotes the number of animals with reproductive problems, and n is the total number of animals evaluated per category.

The proportion of animals with reproductive problems (abortion or the birth of weak live kids) was 48.95% (95% CI: 40.84–57.09) among *N. caninum* seronegative individuals and 84.62% (95% CI: 72.33–94.16) among *N. caninum* seropositive individuals. The Bayesian model indicated a positive and statistically significant effect of *N. caninum* infection on the outcome (β = 1.89; 95% HPD: 0.94–2.95), corresponding to an OR = 6.49 (95% HPD: 1.76–16.11). This implies that the odds of reproductive problems in seropositive animals were nearly 6.5 times higher than in seronegative animals (Figure 5).

### Estimation of the direct, indirect, and total effects

The decomposition of effects was used to evaluate whether *N. caninum* serostatus acts as a mediator in the relationship between geographic zone, age, and reproductive problems (Table 2). The direct effect of Zone 2 relative to Zone 1 had a 95% HPD interval that excluded the null value, indicating a higher likelihood of reproductive problems associated with Zone 2, independent of *N. caninum* serostatus. In contrast, the indirect effect mediated by *N. caninum* was uncertain, as its 95% HPD interval included zero, and the total effect of zone was also not conclusive. These results indicate that differences in the occurrence of reproductive problems between zones are not primarily explained by *N. caninum* infection but rather depend largely on zone-specific factors that are unrelated to this pathogen’s serostatus. In this context, *N. caninum* does not act as a mediator of observed geographic differences but instead functions as an important individual-level risk factor.

**Table 2:** Direct, indirect, and total effects (expressed as log-odds) of location and age on the likelihood of reproductive problems, with *Neospora caninum* serostatus as a mediating variable.

Factors	Indirect	Direct	Total
Zone 1	Reference		
Zone 2	−1.56 (−3.92, 0.48)	1.14 (0.32, 1.96)	−0.42 (−2.85, 1.79)
Age	0.37 (−0.27, 1.08)	0.58 (0.26, 0.91)	0.95 (0.22, 1.72)

Estimates are presented with 95% highest posterior density intervals. “Reference” indicates the baseline category used for contrast estimation.

The direct effect of age on reproductive problems showed a 95% HPD interval that excluded the null value, whereas the indirect effect through *N. caninum* was uncertain because its 95% HPD interval included zero. The total effect of age also showed a 95% HPD interval that excluded the null value, indicating an overall effect of age on the occurrence of reproductive problems. Taken together, this pattern indicates that the age-related increase in reproductive risk is primarily explained by direct mechanisms, with a limited, non-conclusive indirect contribution mediated by *N. caninum*.

## DISCUSSION

### Influence of age and geographical location on the prevalence of *N. Caninum*

Unlike previous studies using simple risk-factor models, this study employs a Bayesian mediation framework to examine direct and indirect pathways linking geography, age, infection status, and reproductive failure. Our findings indicate that *N. caninum* is associated with reproductive failure in Creole goats from southern Peru, with effects varying by age and geographic location. These results were obtained in hyper-arid extensive goat production systems with irregular forage availability, characterized by infectious factors, nutritional constraints, and ecological stressors inherent to the environment.

The prevalence was higher in Zone 1 (Chincha, Pisco, and Ica) than in Zone 2 (Nazca and Palpa), although the Bayesian model analysis was not conclusive. Notably, the seroprevalence observed in our study was markedly greater than that reported in previous studies from other regions of Peru [12, 13]. Previous studies have associated higher *N. caninum* prevalence with more humid environments or lower latitudes [21] and warmer climates [22]. Consistent with this evidence, our results revealed a relatively higher prevalence in the warm, more humid coastal zone, although the difference was not statistically significant, precluding a direct attribution of this difference to climate.

Given that vertical transmission and exposure to dogs are key drivers of infection dynamics, the differences observed in this study may be more plausibly explained by management practices and herd age structure than by environmental factors, although this remains to be confirmed. However, this pattern aligns with findings from other regions, where prevalence has shown marked heterogeneity across agroecological settings and management systems [23, 24]. This pattern suggests that cumulative exposure over time may increase the likelihood of infection. Consistently, earlier research has shown that goats older than 3 years were up to 2.6-fold more likely to test positive than those younger than 1 year [24]. Similarly, another study reported a 2.1-fold increase in seropositivity among older animals compared with younger cohorts [25].

Furthermore, the presence of four to six dogs per herd without an annual deworming program represents an additional epidemiological risk factor by facilitating the shedding of infective oocysts into the environment [7]. Overall, these findings reinforce the role of management-related factors, particularly dog exposure and herd structure, in shaping *N. caninum* transmission patterns in extensive goat production systems.

### Influence of *N. Caninum* serostatus, geographic zone, and age on reproductive disorders

Our findings indicate that *N. caninum* is a direct predictor of reproductive disorders. No significant mediation effect was detected between age, geographic zone, and reproductive outcomes. The absence of significance in the product does not necessarily imply that the mediator exerts no effect [26]. In this study, *N. caninum* seropositivity was directly associated with an increased risk of reproductive failure, independent of age or geographic location.

Previous studies have consistently demonstrated an association between *N. caninum* seropositivity and reproductive disorders, identifying this protozoan as one of the leading causes of abortion in cattle [7]. It has been linked to abortions, stillbirths, and fetal retention in goats [23, 24]. A global meta-analysis estimated that 7%–15% of caprine abortions are attributable to *N. caninum*, with a worldwide prevalence of 7% (95% CI: 2%–12%) in aborted fetuses [23]. Another study reported that seropositive goats had 3.07-fold higher odds of abortion than seronegative animals [11]. In the present study, the observed magnitude was more than twice that value (OR = 6.49); however, the outcome assessed here encompassed reproductive problems rather than abortion alone.

A potential pathogenic mechanism involves the invasion of the placenta by *N. caninum*, particularly of fetal trophoblastic cells, in which active replication induces necrosis and extracellular matrix disruption. These changes may impair maternal–fetal exchange and favor fetal death [27]. The severity of placental damage depends on both parasite strain virulence and gestational stage. Early infections (approximately 10 weeks) often result in resorption or fetal death, whereas later infections may allow the birth of infected but viable offspring [27, 28].

From a spatial perspective, reproductive failures were more frequent in Zone 2, a difference that persisted after adjustment for the mediator, indicating a positive direct effect of geographic zone. Despite the higher seroprevalence of *N. caninum* in Zone 1, reproductive problems were more common in Zone 2, suggesting that parasite exposure alone does not fully account for reproductive outcomes. This finding underscores the need to integrate ecological and nutritional stressors into reproductive risk assessment models.

Environmental conditions may also contribute, as elevated temperatures have been shown to compromise oocyte competence and embryo viability, leading to smaller embryos and reduced transfer success. Both experimental and observational studies have documented lower numbers of transferable embryos and hormonal imbalances under heat stress [29–32]. Although not assessed in males in this study, it is plausible that heat and humidity impair thermoregulation, elevate testicular temperature, and disrupt spermatogenesis, resulting in reduced sperm motility, increased aged acrosomes, and morphological abnormalities [33].

Moreover, the higher frequency of reproductive disorders observed in Zone 2, despite the lower seroprevalence of *N. caninum*, can be attributed to the nutritional and environmental constraints characteristic of this arid ecosystem. Limited forage availability and quality may lead to poor body condition and mineral deficiencies, compromising reproductive efficiency by increasing the risk of abortion and reducing gestational viability. This highlights the pivotal role of nutritional stress and forage scarcity in modulating reproductive performance in extensive systems [34]. In small ruminants, inadequate nutrition is associated with decreased ovulation frequency, altered gonadotropin secretion, reduced fertility, and a higher risk of early embryonic loss [35].

Seasonal fluctuations in forage availability and quality may induce sustained physiological stress in goats, compromising the function of the hypothalamic–pituitary–ovarian axis and maintaining high rates of reproductive failure even under lower infectious pressure [36]. Although body condition and forage quality were not quantitatively assessed, this explanation is biologically plausible, especially considering the shrub-based grazing systems in Zone 2, which are characterized by greater nutritional variability than the alfalfa- and crop residue-based systems in Zone 1 [37].

Taken together, these findings highlight the need for integrated assessments that incorporate nutritional indicators to more accurately differentiate between infectious and non-infectious factors associated with reproductive failure in extensive goat systems.

### Multifactorial nature of reproductive failure and epidemiological implications

Reproductive failure in goats is widely recognized as a multifactorial condition involving infectious agents such as *N. caninum*, nutritional deficiencies, environmental stressors, and management-related factors [9, 21, 22]. In this context, the persistence of reproductive disorders in Zone 2, despite its lower seroprevalence, underscores the importance of non-infectious determinants such as forage quality, body condition, and herd management, which were not quantitatively assessed in the present study but have been shown to strongly influence reproductive performance in extensive systems [6, 9].

Previous studies have identified dogs as a relevant source of *N. caninum* infection in extensive production systems [6, 12]. The results of the present study in Creole goats from southern Peru are consistent with a possible influence of the presence of dogs; however, this factor was not directly evaluated due to the observational nature of the study. Analytical studies have demonstrated that direct contact with dogs significantly increases the probability of seropositivity, with odds ratios up to 4.81, and that interaction with other domestic species, such as cattle, may further contribute to the circulation of parasites in multispecies systems [9, 12, 22]. In this context, the strong association between *N. caninum* seropositivity and reproductive disorders observed in this study (OR = 6.49) reinforces its relevance as an epidemiological indicator of reproductive risk in extensively managed goats.

Finally, there is a high risk of serological cross-reactivity with *Toxoplasma gondii*, particularly in conventional enzyme-linked immunosorbent assays that use soluble tachyzoite antigens, thereby reducing diagnostic specificity [39]. This cross-reactivity varies by test type and cutoff values, and the use of recombinant antigens or complementary serological strategies can mitigate this bias [40]. Therefore, the results should be interpreted as evidence of exposure rather than definitive causality.

### Limitations and recommendations for future research

Although the study employed a Bayesian approach, which is suitable for data analysis with moderate sample sizes, and the number of observations was sufficient to address the main objectives, the study design entails several limitations that should be acknowledged. First, herd selection was not completely random, as sampling was restricted to flocks with a documented history of reproductive problems, and animals with reproductive disorders were preferentially included within these herds. This targeted sampling strategy may introduce selection bias and limit the generalizability of prevalence estimates and associations to the broader goat population.

The study followed a cross-sectional design, with sampling conducted between March and July and no longitudinal follow-up of individual animals. Consequently, the associations observed between *N. caninum* infection, demographic variables, and reproductive problems should be interpreted as epidemiological relationships rather than evidence of causality. Although the sample size allowed the evaluation of the primary associations of interest, it may have limited the ability to detect smaller indirect effects within the mediation framework.

Quantitative assessments of nutritional status, forage quality, body condition, sanitary management, and accurate estimates of dog density or environmental circulation of *N. caninum* at the farm level were not available. Given that these factors may influence both infection dynamics and reproductive performance, their specific contribution to the observed patterns could not be evaluated in this study.

Therefore, future research should prioritize longitudinal study designs that allow the establishment of temporal relationships between infection and reproductive events, the assessment of the role of vertical transmission, and the confirmation of infection through molecular techniques and histopathological examination of fetal and placental tissues. Incorporating nutritional, environmental, and management indicators would further improve the ability to disentangle infectious and non-infectious determinants of reproductive failure in extensive goat production systems.

## CONCLUSION

This study provides the first comprehensive evidence of *N. caninum* infection in Creole goats from the hyper-arid coastal region of southern Peru and demonstrates its strong association with reproductive disorders. The prevalence of *N. caninum* was higher in Zone 1 than in Zone 2; however, reproductive problems were more frequent in Zone 2, suggesting that infection alone does not fully account for the observed reproductive outcomes. Seropositive animals showed markedly higher odds of reproductive disorders (OR = 6.49), confirming *N. caninum* as a significant individual-level risk factor. In addition, age exhibited a direct positive effect on reproductive problems, whereas the mediation analysis indicated that *N. caninum* did not significantly mediate the effects of age or geographic zone.

From a practical perspective, these findings highlight the need for integrated control strategies tailored to extensive goat production systems. Preventive measures such as restricting dog access to kidding areas, feed storage sites, and water sources; ensuring proper disposal of placental and aborted materials and implementing basic health management for farm dogs are essential to reduce environmental contamination and transmission risk. Furthermore, improving nutritional management and forage planning, particularly in arid zones with limited feed resources, is crucial to mitigate non-infectious contributors to reproductive failure.

A key strength of this study lies in the application of a Bayesian mediation framework, which allowed the simultaneous evaluation of direct and indirect effects, providing a more nuanced understanding of the interplay between infection, age, and geographic factors. Additionally, the study addresses a significant epidemiological gap by generating novel data from a previously unstudied agroecological context.

However, several limitations should be considered. The cross-sectional design precludes causal inference, and the targeted sampling of herds with known reproductive problems may limit the generalizability of the findings. Moreover, the absence of quantitative data on nutritional status, body condition, and management practices restricts the ability to fully disentangle infectious from non-infectious determinants of reproductive disorders. Potential serological cross-reactivity with *T. gondii* may also influence diagnostic specificity.

Future research should focus on longitudinal studies to establish temporal relationships between *N. caninum* infection and reproductive outcomes, and to evaluate the role of vertical transmission within herds. The integration of molecular diagnostics, histopathological analyses, and detailed assessments of nutritional and environmental factors will be essential for refining causal inference and improving disease control strategies.

In conclusion, reproductive disorders in Creole goats from southern Peru are multifactorial, with *N. caninum* acting as a significant but not exclusive determinant. Effective mitigation requires a holistic approach that integrates infection control, nutritional management, and improved husbandry practices, particularly in resource-limited extensive production systems.

## DATA AVAILABILITY

The supplementary data can be made available from the corresponding author upon request.

## AUTHORS’ CONTRIBUTIONS

JS and JC-G: Conceptualized and designed the study, developed the methodology, conducted sampling, and drafted the manuscript. MT-S and JV-Z: Designed the study, collected samples, and prepared the initial manuscript. EM-A, VT-S, and EAS: Assisted in the methodology, sample collection and analysis, preliminary results, and manuscript review. JC-L: Data management and preparation of the initial manuscript. DJC: Contributed to data management and formal analysis, interpretation of results, and writing. All authors have read and approved the final version of the manuscript.

## References

[1] Meza-Herrera CA, Navarrete-Molina C, Macias-Cruz U, Arellano-Rodriguez G, De Santiago-Miramontes A, Sariñana-Navarrete MA (2024). Dairy goat production systems: A comprehensive analysis to reframe their global diversity. Animals.

[2] Nair MRR, Sejian V, Silpa MV, Fonsêca VFC, de Melo Costa CC, Devaraj C (2021). Goat as the ideal climate-resilient animal model in tropical environment: Revisiting advantages over other livestock species. Int J Biometeorol.

[3] Sessarego E, Godoy-Padilla D, Mendoza Y, Cruz-Luis J (2025). Phenotypic characterization of the Creole goat in the southern highlands of Peru: A first step toward the sustainable use of a lost zoogenetic resource. Open Vet J.

[4] MIDAGRI. Boletín estadístico mensual “El agro en cifras” (2021). Ministerio de Desarrollo Agrario y Riego, Plataforma del Estado Peruano.

[5] Pereyra WR, Suarez VH, Cardoso N, Gual I, Martínez GM, Capozzo AV (2021). Seroprevalence and risk factors associated with *Neospora caninum* in dairy farms from the Province of Salta, Argentina Rev Argent Microbiol.

[6] Fereig RM, Wareth G, Abdelbaky HH, Mazeed AM, El-Diasty M, Abdelkhalek A (2022). Seroprevalence of specific antibodies to *Toxoplasma gondii*, *N. caninum*, and *Brucella* spp. in sheep and goats in Egypt. Animals.

[7] Dubey JP, Schares G, Ortega-Mora LM (2007). Epidemiology and control of neosporosis and *N. caninum*. Clin Microbiol Rev.

[8] Tagwireyi WM, Garcia Alvarez G, Morar-Leather D, Neves L, Thompson PN (2025). Seroprevalence of *N. caninum* in dairy goats from northern South Africa: A preliminary study. Vet Parasitol Reg Stud Rep.

[9] Vonlaufen N, Müller N, Keller N, Naguleswaran A, Bohne W, McAllister MM (2002). Exogenous nitric oxide triggers *N. caninum* tachyzoite-to-bradyzoite stage conversion in murine epidermal keratinocyte cell cultures. Int J Parasitol.

[10] de Oliveira Junior IM, Mesquita LE dos S, Miranda DNP, Gomes TA, Vasconcelos BKS, Penha LC (2020). Endogenous transplacental transmission of *N. caninum* in successive generations of congenitally infected goats. Vet Parasitol.

[11] Araújo Rodrigues A, Silva Reis S, Lima de Sousa M, da Silva Moraes E, Garcia JL, Costa Nascimento TV (2020). A systematic literature review and meta-analysis of risk factors for *N. caninum* seroprevalence in goats. Prev Vet Med.

[12] Aranda M, Pinedo R, Abad-Ameri D, Chávez A (2023). Seroprevalence of *Neospora caninum* in goats (*Capra hircus*) from the Piura region, Peru [Seroprevalencia de *N. caninum* en cabras (*Capra hircus*) de la región Piura, Perú]. Rev Inv Vet Perú.

[13] Tafur Gutiérrez L, Alva G, Godoy DJ, Frías H, Arista MA, Bardales W (2025). Impact of production practices and sanitary management on the prevalence of *N. caninum* and bluetongue virus in Creole goats from the tropical dry forest of Utcubamba, Peru. Am J Vet Res.

[14] Chemineau P, Malpaux B, Brillard JP, Fostier A (2007). Seasonality of reproduction and production in farm fishes, birds and mammals. Animal.

[15] ESRI. ArcMap (2016). Environmental Systems Research Institute.

[16] SENAMHI. Descarga de datos meteorológicos Servicio Nacional de Meteorología e Hidrología del Perú.

[17] Innovative Diagnostics. ID Screen®*N. caninum* Competition (2024). Innovative Diagnostics.

[18] Rafiq M, Khan NU, Khan I, Ahmad M, Bibi A, Ben Said M (2024). Evaluating prevalence, risk factors, and diagnostic techniques for Cryptosporidium infection in goats and surrounding water sources. Front Vet Sci.

[19] Bürkner PC (2017). brms: An R package for Bayesian multilevel models using Stan. J Stat Softw.

[20] R Core Team. R: A language and environment for statistical computing (2024). R Foundation for Statistical Computing.

[21] Mendoza-Morales LF, Lagorio V, Corigliano MG, Sánchez-López E, Ramos-Duarte VA, Clemente M (2022). Neosporosis in sheep: A systematic review and meta-analysis of global seroprevalence and related risk factors. Acta Trop.

[22] Rinaldi L, Fusco G, Musella V, Veneziano V, Guarino A, Taddei R (2005). *N. caninum* in pastured cattle: Determination of climatic, environmental, farm management and individual animal risk factors using remote sensing and geographical information systems. Vet Parasitol.

[23] Nayeri T, Sarvi S, Moosazadeh M, Daryani A (2022). The global prevalence of *N. caninum* infection in sheep and goats that had an abortion and aborted fetuses: A systematic review and meta-analysis. Front Vet Sci.

[24] Varaschin MS, Guimarães AM, Hirsch C, Mesquita LP, Abreu CC, Rocha CMBM (2011). Factors associated to seroprevalence of *Neospora caninum* and *Toxoplasma gondii* in caprine herds in southern Minas Gerais state, Brazil [Fatores associados àsoroprevalência de *N. caninum* e *Toxoplasma gondii* em rebanhos caprinos na região sul de Minas Gerais]. Pesq Vet Bras.

[25] Araújo Rodrigues A, Silva Reis S, da Silva Moraes E, do Nascimento Souza Filho JG, dos Santos Reis MH, Martins TA (2021). Seroprevalence and risk factors for *N. caninum* and *Toxoplasma gondii* in goats of Maranhão State, Brazil. Vet Parasitol Reg Stud Rep.

[26] MacKinnon DP, Lockwood CM, Hoffman JM, West SG, Sheets V (2002). A comparison of methods to test mediation and other intervening variable effects. Psychol Methods.

[27] Jiménez-Pelayo L, García-Sánchez M, Collantes-Fernández E, Regidor-Cerrillo J, Horcajo P, Gutiérrez-Expósito D (2020). Crosstalk between *N. caninum* and the bovine host at the maternal–foetal interface determines the outcome of infection. Vet Res.

[28] Williams DJ, Guy CS, McGarry JW, Guy F, Tasker L, Smith RF (2000). *N. caninum*-associated abortion in cattle: The time of experimentally induced parasitemia during gestation determines foetal survival. Parasitology.

[29] Adjassin JS, Assani AS, Bani AA, Sanni Worogo HS, Adégbeïga Alabi CD, Comlan Assogba BG (2022). Impact of heat stress on reproductive performances in dairy goats under tropical sub-humid environment. Heliyon.

[30] Çizmeci SÜ, Dinç DA, Yesilkaya OF, Çiftçi MF, Takcı A, Bucak MN (2022). Effects of heat stress on oocyte number and quality and *in vitro* embryo production in Holstein heifers. Acta Sci Vet.

[31] Kasimanickam R, Kasimanickam V (2021). Impact of heat stress on embryonic development during first 16 days of gestation in dairy cows. Sci Rep.

[32] Ratchamak R, Ratsiri T, Chumchai R, Boonkum W, Chankitisakul V (2021). Relationship of the temperature–humidity index with ovarian responses and embryo production in superovulated Thai–Holstein crossbreds under tropical climate conditions. Vet Sci.

[33] Krishnan G, Bagath M, Pragna P, Vidya MK, Aleena J, Archana PR, Payan Carreira R (2017). Mitigation of the heat stress impact in livestock reproduction. Theriogenology.

[34] Mellado M, Pastor FJ (2006). Aborto no infeccioso en caprinos. Ciência Anim Bras.

[35] Robinson JJ, Ashworth CJ, Rooke JA, Mitchell LM, McEvoy TG (2006). Nutrition and fertility in ruminant livestock. Anim Feed Sci Technol.

[36] Martin GB, Rodger J, Blache D (2004). Nutritional and environmental effects on reproduction in small ruminants. Reprod Fertil Dev.

[37] National Research Council (2007). Nutrient requirements of small ruminants.

[38] Akhtar M, Khandoker M, Akter T (2023). Effect of age on follicular dynamics of goat ovaries. Bangladesh J Anim Sci.

[39] Gondim LFP, Mineo JR, Schares G (2017). Importance of serological cross-reactivity among *Toxoplasma gondii*, *Hammondia* spp., Neospora spp., *Sarcocystis* spp. and *Besnoitia besnoiti*. Parasitology.

[40] Hebbar BK, Roy M, Mitra P, Chavhan K, Chaudhari S, Shinde S (2022). Seroprevalence, risk factors, and serological cross-reactivity for diagnosis of *Toxoplasma gondii* and *N. caninum* infections in goats in India. Microb Pathog.

